# Bilateral Spontaneous Dissection of the Superficial Femoral Arteries (SFAs): A Case Report

**DOI:** 10.7759/cureus.91849

**Published:** 2025-09-08

**Authors:** Wasifuddin Syed, Khudheeja A Ahmed, Juwayria A Ahmed, Mohammed Habeeb Ahmed

**Affiliations:** 1 College of Medicine, Edward Via College of Osteopathic Medicine, Monroe, USA; 2 College of Osteopathic Medicine, Touro University California, Vallejo, USA; 3 Department of Research, KAAJ Healthcare, San Jose, USA; 4 Department of Cardiology, KAAJ Healthcare, San Jose, USA

**Keywords:** bilateral, claudication, ischemia, spontaneous arterial dissection, superficial femoral artery

## Abstract

Spontaneous arterial dissection regarding the superficial femoral artery (SFA) is known to be an uncommon finding, and this clinical entity, when noted bilaterally, is exceptionally rare. Diagnosis is typically achieved by imaging modalities such as angio-tomography (angio-CT) or arteriography.

This case report involves a 78-year-old male patient with a history of hypertension (HTN), cardiovascular disease, and arrhythmias who presented with leg pain and aches. A peripheral angiogram diagnosed spontaneous bilateral dissection of the SFA. This case adds to the literature by documenting this rare phenomenon, highlighting the need for clinical awareness of bilateral spontaneous SFA dissection in differential diagnoses for bilateral lower extremity ischemia, even in the setting of no trauma or traditional risk contributors.

## Introduction

Spontaneous arterial dissection of the superficial femoral artery (SFA) is known to be a rare vascular condition, with only a limited number of documented cases in existing literature [1]. This phenomenon involves a tear within the arterial wall’s linings of the tunica intima or tunica media, occurring with no identifiable trigger or preceding trauma [1,2]. As a result, blood accumulates within the vessel wall, producing serious consequences [2]. It is often observed in individuals with underlying risk factors, including connective tissue disorders, pregnancy, atherosclerotic development, or a recent history of a high level of physical exertion [1,3,4]. Clinically, patients typically present with acute limb symptoms, including significant pain, numbness, and reduced peripheral pulses [1]. The breakdown of the arterial wall and subsequent intramural hematoma can obstruct narrower vessels, leading to ischemic complications [2]. The true lumen may be blocked, and the hematoma can result in emboli that obstruct vessels distally [2]. This case adds to the literature by describing a patient with bilateral spontaneous SFA dissection and outlining its clinical presentation, diagnostic workup, and imaging findings.

## Case presentation

A 78-year-old male patient with a past medical history of hypertension (HTN), dyslipidemia, cardiomyopathy, non-obstructive coronary artery disease (CAD), peripheral artery disease (PAD), and arrhythmias (status post pacemaker) presented to the office with progressive bilateral lower extremity pain. He reported aching and cramping in both calves, more so at night, sometimes waking him from sleep. He also reported difficulty ambulating the amount of distance he used to. His current medications include Eliquis (5 mg QD), Carvedilol (25 mg BID), Lisinopril (40 mg QD), Ranolazine (500 mg), Isosorbide (30 mg), and Amlodipine (10 mg).

**Figure 1:**
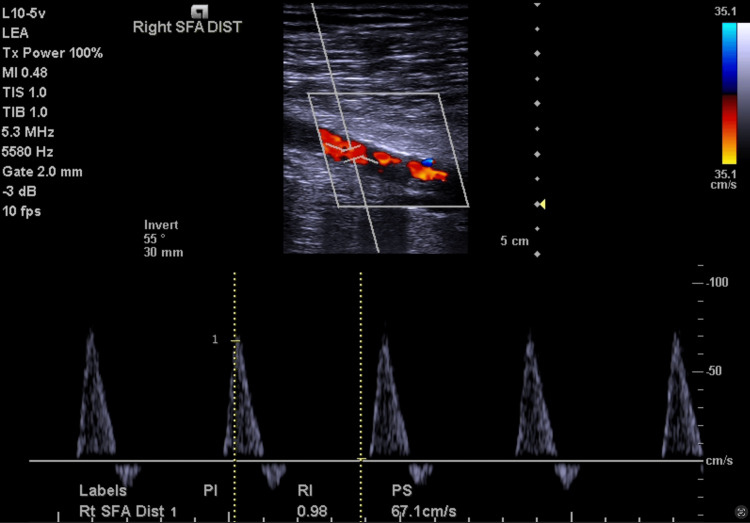
Arterial ultrasound revealed biphasic waveforms on the right side with moderate infrapopliteal disease and decreased velocity

**Figure 2:**
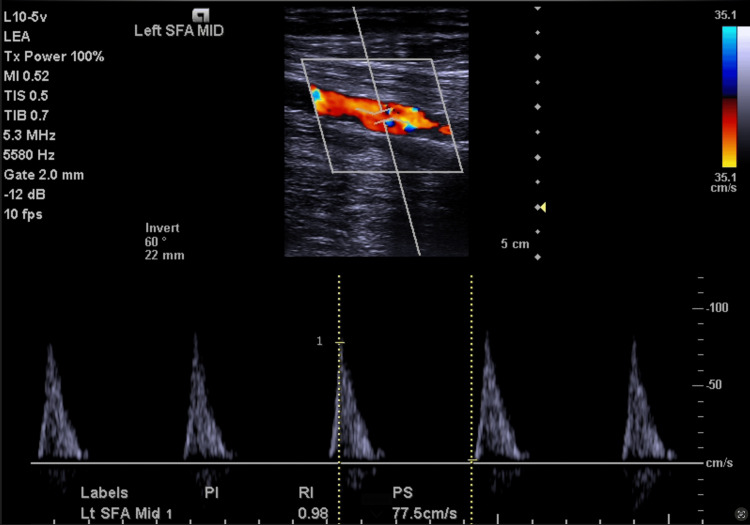
Arterial ultrasound revealed monophasic to biphasic waveforms on the left side with moderate infrapopliteal disease and decreased velocity

**Figure 3:**
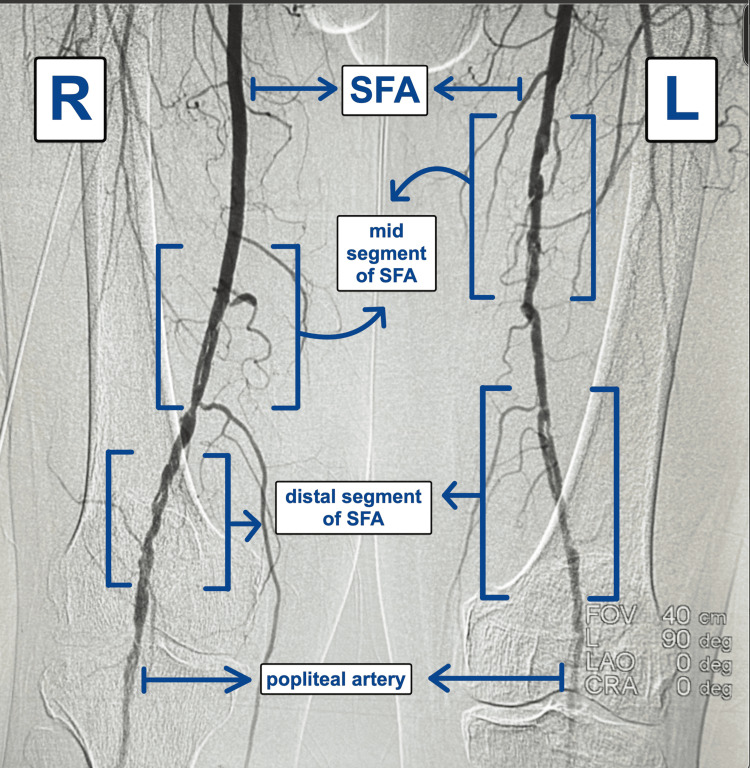
This figure shows the spontaneous dissection in the bilateral superficial femoral arteries (SFAs) in the mid and distal segments

Arterial ultrasound performed in October 2024 revealed biphasic waveforms on the right side (Figure 1) and monophasic to biphasic waveforms on the left (Figure 2) with moderate infrapopliteal disease and decreased velocities seen bilaterally, with the left leg being worse than the right. Given worsening symptoms of decreasing intermittent claudication distance with increased frequency and known PAD, as well as the abnormal ultrasound findings, peripheral angiography was performed in December 2024. The ultrasound was not suggestive of any dissection. Angiographic evaluation revealed diffuse disease of the right SFA with spontaneous dissection in its mid and distal segments (Figure 3). Collateral flow was observed from the profunda femoris artery to the infrapopliteal vessels, suggestive of obstructed flow to the infrapopliteal vessels (Figure 3). Similar findings were seen on the left, with spontaneous dissection in the mid and distal segments of the SFA (Figure 3). These findings were consistent with bilateral dissection of the SFAs (Figure 3), so this was diagnosed and deemed to be a spontaneous dissection. There were no procedural complications throughout. The patient has not yet undergone intervention. After counseling regarding the rare diagnosis of spontaneous dissection, the family chose to defer angioplasty, and outpatient vascular evaluation and treatment planning were initiated.

## Discussion

Spontaneous dissection of peripheral arteries is an infrequent occurrence, and the iliac artery is the most commonly affected site when it does occur [1]. In contrast, spontaneous bilateral dissection of the superficial femoral arteries (SFA) is exceedingly rare [1]. Several case reports in the literature highlight the variable presentation and management approaches of such vascular dissections.

Rahmani et al., in 2002, describe an instance of spontaneous dissection regarding the superficial femoral artery in a distal segment in a 46-year-old male individual [1,5]. This patient’s clinical presentation included acute left calf pain treated by resection and reconstruction using an interposition graft [1,5]. One case by Rajagopalan et al. described a 46-year-old male patient without a past medical history of trauma, smoking, or family history who presented with intense pain in the left leg [6]. Following angiographic findings led to the diagnosis of left superficial femoral arterial dissection, addressed with an “SFA to peroneal bypass” using the left long saphenous vein, with a full recovery in the left leg’s function [6].

Spontaneous femoral arterial dissection has been linked to pregnancy, connective tissue disorders, decreased production of alpha-1-antitrypsin, genetic or congenital factors, and atherosclerotic development [1,3,4]. Additional contributing factors are trauma and aneurysms [6]. These factors are believed to compromise arterial wall integrity, predisposing patients to dissection [1].

Spontaneous arterial dissection can be identified through imaging modalities such as grayscale ultrasonography combined with color Doppler [1]. On Doppler evaluation, the presence of two distinct flow pathways indicates the true and false lumens [1]. False lumens can have flow in the opposite direction of true flow and can include thrombotic deposits [1]. Further evaluation with angiography can delineate areas of luminal narrowing and irregularity, helping clinicians pinpoint affected segments and guide therapeutic interventions such as angioplasty [1]. Intravascular ultrasound (IVUS) offers a more detailed understanding of vascular wall layers, providing insight into the extent and nature of arterial abnormalities [1].

A key differential diagnosis for this clinical entity is pseudoaneurysm, which typically arises in the context of trauma or inflammatory and neoplastic factors [1]. Pseudoaneurysms appear as perfused outpouches connected to the lumen [1,7]. B-mode ultrasound serves as a primary imaging modality for detecting these lesions [1]. Color Doppler imaging provides hemodynamic information that can assist in distinguishing this phenomenon from spontaneous arterial dissections [1].

There is no standardized treatment protocol for this condition, as management protocols depend on the particularities and clinical presentation of each case [1]. Reported approaches in the literature include conservative treatment, endovascular intervention, and surgical repair [1]. Among these, endovascular techniques are frequently preferred as first-line therapy and have been shown to result in successful revascularization outcomes [1].

This case adds to the limited body of literature present as of now on spontaneous bilateral superficial femoral artery dissection, a rare vascular event with few documented occurrences. This case underscores the potential for bilateral dissection in elderly patients with cardiovascular comorbidities but no known connective tissue disorder or recent trauma. The patient presented with symptoms of PAD, including nocturnal cramping and intermittent claudication with decreasing claudication distance, emphasizing the importance of considering spontaneous arterial dissection in the differential diagnosis of worsening claudication. The patient’s history of HTN, CAD, and PAD further highlights the need for clinical suspicion in these patient populations.

## Conclusions

Spontaneous arterial dissection of the superficial femoral artery is a very uncommon vascular finding. This report presents the case of a 78-year-old male patient who presented with leg aches and pains, nocturnal cramping, and decreased ambulation. His past medical history included hypertension, coronary artery disease, peripheral artery disease, and arrhythmias with status post pacemaker implantation. He was diagnosed with spontaneous bilateral dissection of the SFA with angiographic imaging. We present this case to contribute to the limited literature on this rare condition. Early clinical recognition of the presenting symptoms, regardless of the presence of comorbidities or trauma history, is essential for timely diagnosis and management. By documenting this rare case of SFA spontaneous bilateral dissection, we aim to contribute to the understanding of its presentation and reinforce the importance of considering this clinical entity across a broad range of patient populations.
